# 

**Published:** 1856-01

**Authors:** 


### RECEIPTS FOR THE REGISTER.

| | | | |
|---|---|---|---|
| Dr. B. F. Dewey | $2 00 | Dr. W. C. Barnard | $3 00 |
| Dr. A. Berry | 3 00 | Dr. Jas, Clark | 6 00 |
| Dr. McQuean | 2 00 | Dr. —— Pleasants | 4 00 |
| Dr. S. McClure | 3 00 | Dr. A. J Reeve | 3 00 |
| Dr. —— Gilmore | 3 00 | Dr. J. W, Wortman | 2 00 |
| Dr. J. W. Clark | 3 00 | Dr. H. A. Hartzell | 75 |

